# Supplementary material for: Vortex rectenna powered by environmental fluctuations
Source: Nat Commun. 2018 Nov 22;9:4922. doi: 10.1038/s41467-018-07352-1 (PMC6250711; doi:10.1038/s41467-018-07352-1)
Supplement: Supplementary file 1 — Supplementary Information [file 41467_2018_7352_MOESM1_ESM.pdf]

Supplementary Information

Vortex rectenna powered by environmental fluctuations

Lustikova et al.

Table of Contents

Supplementary Note 1. Theoretical model for vortex flow rectification **3**

1.1 Ginzburg-Landau free energy for a single vortex 3

1.2 Vortex energy with a ferromagnetic substrate 6

1.3 Vortex nucleation asymmetry 7

1.4 Consideration of sample length scales 9

Supplementary Note 2. Additional experimental results **11**

2.1 Angular dependence of d.c. voltages 11

2.2 Estimate of the d.c. voltage generated by rectification of environmental noise 13

2.3 Temporal evolution of d.c. voltage in MoGe|YIG with superconducting magnet in persistent mode. 14

2.4 Change in d.c. voltage generated in MoGe|YIG by switching of the superconducting magnet between driven and persistent modes. 15

2.5 Magnetic field dependence of d.c. voltages generated in MoGe|YIG for various in-plane temperature gradients. 16

2.6 Magnetic field dependence of the resistivity, Δ_{2f}, and generated d.c. voltages in MoGe|SiO_2(10 nm)|YIG at selected temperatures. 17

2.7 Comparison of d.c. voltages generated in MoGe|SiO_2, MoGe|$Y_3Al_5O_{12}$(YAG) and MoGe|YIG 18

2.8 Magnetic field dependence of d.c. voltages in MoGe|YIG at temperatures above 6 K 19

2.9 Influence of SiO_2 thickness on d.c. voltages in MoGe|SiO_2|YIG 20

2.10 Effect of the shape and size of electric contacts on d.c. voltages in MoGe|YIG 21

2.11 Effect of shielding and grounding on d.c. voltages in MoGe|YIG 22

2.12 Influence of the magnet power supply noise on the d.c. voltage in MoGe|YIG 23

2.13 Diagram of the rectenna device 24

Supplementary References **25**

Supplementary Note 1.
Theoretical model for vortex flow rectification

1.1 Ginzburg-Landau free energy for a single vortex

First, we summarize the vortex energy of a single vortex string based on the Ginzburg-Landau (GL) theory for a type II superconductor. (See Supplementary Reference 1.)

The GL free energy is given by

$$F_{\rm GL} = \int d^3r \left[\frac{\hbar^2}{2m^*} \left| \left(\boldsymbol{\nabla} - i\frac{e^*}{\hbar c}\mathbf{A} \right) \psi \right|^2 + \alpha|\psi|^2 + \frac{\beta}{2}|\psi|^4 + \frac{1}{8\pi}\mathbf{H}^2 \right], \tag{1}$$

where ψ is the order parameter corresponding to Cooper pairs with the effective mass m^* and charge e^*, and α and β are the (material-dependent) GL coefficients. Here, we consider the London limit, where the order parameter is given by $\psi(r) = \Delta e^{i\theta(r)}$ with a constant $\Delta = \sqrt{|\alpha|/\beta}$. In this limit, the GL free energy becomes

$$F_{\rm GL} = \int d^3r \left[\frac{\hbar^2 \Delta^2}{2m^*} \left(\boldsymbol{\nabla}\theta - \frac{e^*}{\hbar c}\mathbf{A} \right)^2 + \frac{1}{8\pi}\mathbf{H}^2 - \frac{|\alpha|^2}{2\beta} \right]. \tag{2}$$

The minimization of the free energy leads to the GL equations in the London limit,

$$\boldsymbol{\nabla} \cdot \left(\boldsymbol{\nabla}\theta - \frac{e^*}{\hbar c}\mathbf{A} \right) = 0, \quad \text{and} \quad \frac{1}{4\pi}\left(\boldsymbol{\nabla} \times \mathbf{H}\right) - \frac{2e^*\hbar\,\Delta^2}{m^* c}\left(\boldsymbol{\nabla}\theta - \frac{e^*}{\hbar c}\mathbf{A} \right) = 0. \tag{3}$$

Using the second equation, we can rewrite the GL free energy:

$$\begin{aligned} F_{\rm GL} &= \frac{1}{8\pi} \int d^3r \left[\mathbf{H}^2 + \lambda_{\rm L}^2 (\boldsymbol{\nabla} \times \mathbf{H})^2 \right] \\ &= \frac{1}{8\pi} \int d^3r\, \mathbf{H} \cdot \left[\mathbf{H} + \lambda_{\rm L}^2 \left(\boldsymbol{\nabla} \times \boldsymbol{\nabla} \times \mathbf{H} \right) \right] + \frac{\lambda_{\rm L}^2}{8\pi} \int_\Sigma d^2r \, \left[\mathbf{H} \times (\boldsymbol{\nabla} \times \mathbf{H}) \right]_\perp , \end{aligned} \tag{4}$$

where the London penetration depth is given by $\lambda_{\rm L}^2 = m^* c^2 / 4\pi\, e^{*2} \Delta^2$. We have performed the partial integration for the second equality, which leads to the surface integral on the boundary Σ. Note that this form of the GL free energy is applicable to the small ξ limit, where ξ is the coherence length.

For a vortex structure, we consider a winding configuration of the phase $\theta(r)$,

$$\boldsymbol{\nabla} \times \boldsymbol{\nabla}\theta = 2\pi\, \mathbf{d}_{\rm v}\, \delta^2(r), \tag{5}$$

where $\mathbf{d}_{\rm v}$ is the unit vector along the vortex string, and the delta function has the support on the vortex core. From the GL equations [Supplementary Eq. (3)], we can

obtain the vortex equation in the London limit,

$$\nabla \times \nabla \times \mathbf{H} + \frac{1}{\lambda_{\rm L}^2}\mathbf{H} = \left(\frac{\Phi_0}{\lambda_{\rm L}^2}\right) \mathbf{d}_{\rm v}\, \delta^2(r), \tag{6}$$

which leads to a vortex string solution with the quantum flux $\Phi_0 = hc/e^*$. Now, we can evaluate the vortex energy of the vortex string solution using the GL free energy. Inserting the vortex equation into Supplementary Eq. (4), the bulk contribution becomes

$$F_{\rm GL} \simeq \frac{1}{8\pi}\Phi_0 \int_C ds\; \mathbf{H}_{\rm V} \cdot \mathbf{d}_{\rm v}, \tag{7}$$

where $\mathbf{H}_{\rm V}$ is the magnetic field at the vortex core, and the line integral is performed along the vortex string which generally has a curved profile, C. The general discussion on the vortex energy shows that the surface contribution in the GL free energy [Supplementary Eq. (4)] can be almost ignored, in comparison with the bulk contribution (Supplementary Reference 2).

So far, we have ignored the effect of the external magnetic field $\mathbf{H}_0$ ($H_0 = |\mathbf{H}_0|$). In order to include the contribution from the external magnetic field, we should consider the Gibbs free energy $G_{\rm GL}$ of the GL theory:

$$G_{\rm GL} = F_{\rm GL} - \frac{1}{4\pi}\int d^3r\; \mathbf{H}_0 \cdot \mathbf{H}, \tag{8}$$

where $F_{\rm GL}$ is given by Supplementary Eq. (4). With an external magnetic field, $\mathbf{H}_0$, along the z direction, the magnetic field in the superconductor has the vortex component and the Meissner component near the interface:

$$\mathbf{H} = \mathbf{H}_{\rm V} + \mathbf{H}_{\rm M} \quad \text{with} \quad \mathbf{H}_{\rm M} = \mathbf{H}_0 \exp\left(-\frac{y}{\lambda_{\rm L}}\right), \tag{9}$$

where the coordinate y (>0) parameterizes the direction perpendicular to the interface. The Meissner component $\mathbf{H}_{\rm M}$ satisfies the equation,

$$\nabla \times \nabla \times \mathbf{H}_{\rm M} + \frac{1}{\lambda_{\rm L}^2}\mathbf{H}_{\rm M} = 0. \tag{10}$$

Inserting $\mathbf{H} = \mathbf{H}_{\rm V} + \mathbf{H}_{\rm M}$ into Supplementary Eq. (8) and using the Supplementary Eqs. (6) and (10), we obtained the general form of the free energy of the vortex string:

$$G_{\rm GL} \simeq \frac{1}{4\pi}\,\Phi_0 \int_C ds\; \left(\frac{\mathbf{H}_{\rm V}}{2} - \mathbf{H}_0 + \mathbf{H}_{\rm M}\right) \cdot \mathbf{d}_{\rm v}. \tag{11}$$

With a large external magnetic field parallel to the interface (in the z-direction), the

vortex string tends to be aligned along the magnetic field. However, the vortex energy is proportional to its length, and the nucleation of such a rectilinear vortex string costs large energy. Therefore, it is natural to consider that a vortex string is nucleated with its endpoints at an interface and the perpendicular intersection, which satisfies the boundary condition of the GL theory (Supplementary Ref. 3). Therefore, one can assume that a vortex string is dominantly nucleated in a semicircle form (Supplementary Ref. 2) at the 2D interface (Supplementary Fig. 1). With a semicircle vortex of the radius ρ_v, the line integral can be performed with the constant H_V, and the final form of the vortex energy becomes

$$G_\mathrm{GL} \simeq \left(\frac{\Phi_0\, H_\mathrm{V}\, \lambda_\mathrm{L}}{4\pi}\right)\left(\frac{\rho_\mathrm{v}}{\lambda_\mathrm{L}}\right)\left[\left(\frac{\pi}{2} - \frac{2\,H_0}{H_\mathrm{V}}\right) + \frac{2\,H_0}{H_\mathrm{V}}\int_0^{\frac{\pi}{2}} d\theta \cos\theta \exp\left(-\frac{\rho_\mathrm{v}}{\lambda_\mathrm{L}}\cos\theta\right)\right]. \quad (12)$$

The value of $H_\mathrm{V} = |\mathbf{H}_\mathrm{V}| \simeq \Phi_0/\lambda_\mathrm{L}^2$ can be calculated from the London equation [Supplementary Eq. (6)], and this value depends only on the bulk properties of the superconductor.

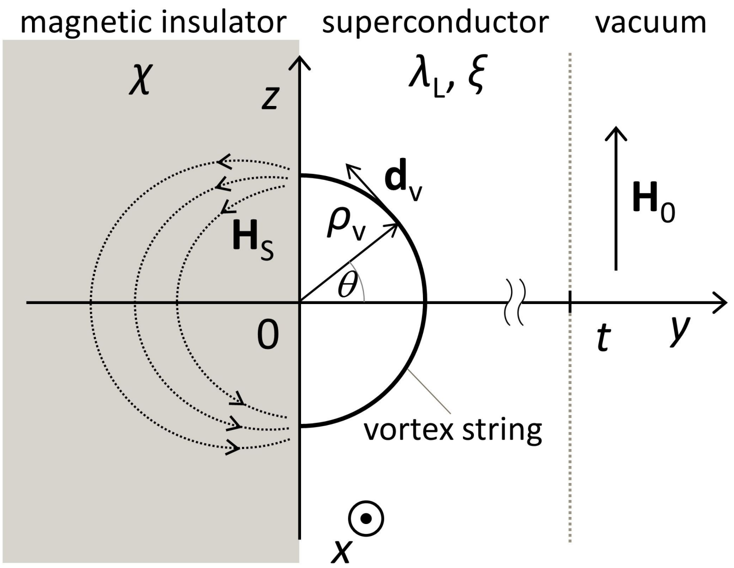

Supplementary Figure 1: A vortex string in the form of a semicircle of the radius ρ_v near a flat interface of a superconductor. The superconductor occupies the space $0 \leq y \leq t$ and is characterized by the magnetic-field penetration depth λ_L and the coherence length ξ. The half-space $y \leq 0$ is occupied by a magnetic insulator with the magnetic susceptibility χ. An external magnetic field $\mathbf{H}_0$ is applied in the z-direction. Stray magnetic fields $\mathbf{H}_\mathrm{S}$ emanate from the end points of vortex strings. $\mathbf{d}_\mathrm{v}$ is a unit vector along the vortex string at an angle θ measured from the positive z axis in the yz-plane.

1.2 Vortex energy with a ferromagnetic substrate

Here, we consider the stray magnetic field in the insulating region ($y < 0$ in Supplementary Fig. 1), which is created by vortex strings terminating at the interface. In this region, the stray magnetic field $\mathbf{H}_\mathrm{S}$ satisfies the static Maxwell equation without a current,

$$\boldsymbol{\nabla} \cdot \mathbf{H}_\mathrm{S} = 0, \quad \boldsymbol{\nabla} \times \mathbf{H}_\mathrm{S} = 0. \tag{13}$$

The nontrivial solution comes from the boundary condition on the surface $y = 0$, due to the continuity of the magnetic field,

$$\mathbf{H}_\mathrm{S} = \mathbf{H}_\mathrm{V} \quad \text{at} \quad y = 0. \tag{14}$$

In the small ρ_v limit, $\rho_\mathrm{v} \ll r = \sqrt{x^2 + y^2 + z^2}$, the solution for $\mathbf{H}_\mathrm{S}$ is approximately given by the dipole magnetic field emanating from the endpoints of the semicircle vortex (Supplementary Ref. 2):

$$\mathbf{H}_\mathrm{S} = -\boldsymbol{\nabla} U_\mathrm{d}, \quad U_\mathrm{d} = P \frac{z}{r^3}, \tag{15}$$

where the magnetic dipole moment P is given by

$$P = 2\, \rho_\mathrm{v}\, \Phi_0. \tag{16}$$

This stray magnetic field $\mathbf{H}_\mathrm{S}$ penetrates into the ferromagnetic substrate, and induces the magnetic energy due to the magnetization $\mathbf{M}$ in the ferromagnet (FM),

$$F_\mathrm{mag} = -\int_{y<0} d^3r\ \mathbf{H}_\mathrm{S} \cdot \mathbf{M}. \tag{17}$$

With a large external magnetic field, $\mathbf{H} = (0, 0, H_0)$, the magnetization in FM is aligned along the magnetic field, $\mathbf{M} = (0, 0, M_0)$, with the saturation magnetization $M_0 = \chi_\parallel H_0$. Including the vortex stray field, the magnetic energy is given by

$$F_\mathrm{mag} = F_{\mathrm{S}\parallel} + F_{\mathrm{S}\perp} = -M_0 \int_{y<0} d^3r\ H_{\mathrm{S}\parallel} - \int_{y<0} d^3r\ \mathbf{H}_{\mathrm{S}\perp} \cdot \delta\mathbf{M}_\perp, \tag{18}$$

where the transverse magnetization is given by $\delta\mathbf{M}_\perp = \chi_\perp \mathbf{H}_{\mathrm{S}\perp}$. With the vortex stray field [Supplementary Eq. (15)], the first term can be evaluated as

$$F_{\mathrm{S}\parallel} \simeq M_0\, \Phi_0\, \rho_\mathrm{v} \left(\frac{D}{L}\right), \tag{19}$$

in the large L limit, where L and D are the length (in the z direction) and thickness

(in the y direction) of the YIG substrate, respectively. In our experimental setup, this contribution is negligibly small due to $D/L \ll 1$. In the large L limit, the leading contribution in the second term of Supplementary Eq. (18) becomes

$$F_{\mathrm{S}\perp} \simeq -\chi_\perp \Phi_0^2 \left(\frac{\rho_{\mathrm{v}}^2}{\xi^3}\right) = -\left(\frac{\Phi_0 H_{\mathrm{V}} \lambda_{\mathrm{L}}}{4\pi}\right) 4\pi\, \chi_\perp \kappa^3 \left(\frac{\rho_{\mathrm{v}}}{\lambda_{\mathrm{L}}}\right)^2 \tag{20}$$

with a coefficient of the order of unity, where the coherence length ξ specifies the size of the vortex core and provides a cutoff parameter in the integration and $\kappa = \lambda_{\mathrm{L}}/\xi$. Note that this transverse contribution does not depend on the size of the magnetic substrate. This term allows the vortex strings to feel the magnetic susceptibility at the surfaces or the interfaces via the nucleation process.

1.3 Vortex nucleation asymmetry

For simplicity, we assume that the vortex flow density induced by a bias current in the vortex liquid phase is governed by the nucleation process of vortex strings at the interfaces. Due to the high mobility of vortex strings in the vortex liquid phase, vortex strings propagate freely in the bulk under the driving force, and their flow is determined by the nucleation rate at the interface from which they are driven away. (See Supplementary Ref. 4 for a similar argument on magnetic skyrmions.)

From the discussion so far, the semicircle vortex energy with the YIG substrate and the external magnetic field is given by $F_{\mathrm{V}}(\rho_{\mathrm{v}}) = G_{\mathrm{GL}} + F_{\mathrm{S}\perp}$. The vortex energy is a function of the radius ρ_{v} of the semicircle, and plotted below with a set of typical parameters.

As seen from Supplementary Fig. 2, the vortex energy has an energy barrier around ρ_0, which is an analogue of the surface barrier for the rectilinear vortex (Supplementary Ref. 5). The nucleation rate P of a semicircle vortex string at the interface is given by the standard nucleation formula $P \simeq \Omega\, e^{-\Delta F/k_{\mathrm{B}}T}$(Supplementary Ref. 6), where ΔF is the height of the vortex energy barrier, Ω is the so-called frequency prefactor, and k_{B} is the Boltzmann constant. The difference in the height of the energy barrier between the YIG and vacuum interfaces is given by the contribution of the magnetization,

$$\Delta F|_{\mathrm{YIG}} - \Delta F|_{\mathrm{vac}} = F_{\mathrm{S}\perp}(\rho_0), \tag{21}$$

and this leads to the asymmetry of the nucleation rate of the semicircle vortex :

$$\Delta P = P_{\mathrm{YIG}} - P_{\mathrm{vac}} \simeq -\Omega \frac{F_{\mathrm{S}\perp}(\rho_0)}{k_{\mathrm{B}}T}, \tag{22}$$

where the small ρ_0 limit (*i.e.* the small $F_{\mathrm{S}\perp}$ limit) is considered. With the form of the magnetic energy [Supplementary Eq. (20)], the asymmetry of the nucleation rate is

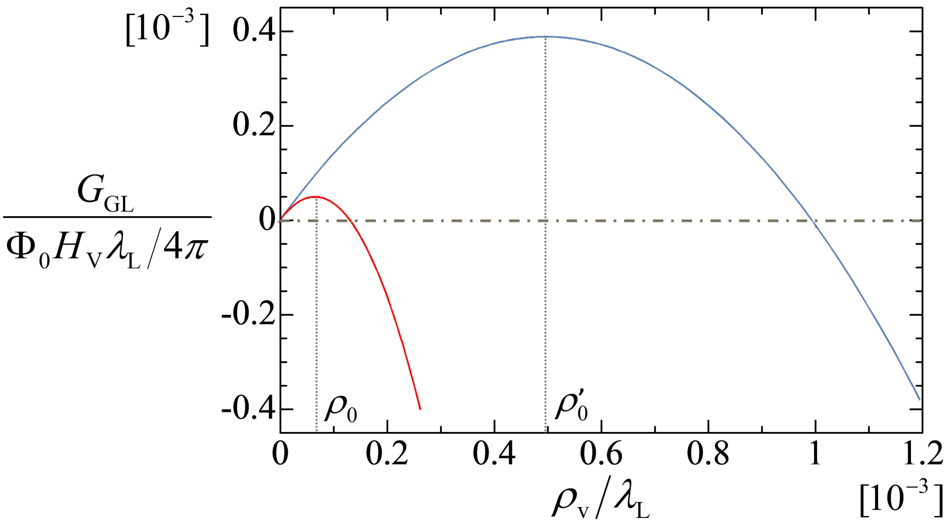

Supplementary Figure 2: Blue line is a plot of the (normalized) vortex energy $G_{\rm GL}$ as the function of the semicircle radius $\rho_{\rm v}/\lambda_{\rm L}$ with $H_0/H_{\rm V} = 1000$. Red line is a plot of the vortex energy including the magnetic energy from the magnetization, $F_{\rm V} = G_{\rm GL} + F_{\rm S\perp}$, with $H_0/H_{\rm V} = 1000$, $4\pi\chi_\perp = 0.01$, and $\kappa = 100$.

given by

$$\Delta P = P_{\rm YIG} - P_{\rm vac} \simeq \chi_\perp \, \Phi_0^2 \, \Omega \left(\frac{\rho_0^2}{k_{\rm B} T \xi^3} \right). \tag{23}$$

Note that in equilibrium without a background current, the asymmetric nucleation rate does not produce a macroscopic vortex flow, since the effect of ΔP is compensated by the backflow (Supplementary Ref. 7) due to the resultant accumulation of vortex strings.

In the presence of the background current $\mathbf{I}$ in the superconductor, vortex strings feel the Lorentz force $\mathbf{f}_{\rm L} \propto \mathbf{I} \times (\mathbf{H}_{\rm V}/c)$. Under the current $-\mathbf{I}_0 = (-I_0, 0, 0)$, the Lorentz force generates the vortex flow $\mathbf{J}_{\rm V}$ from the YIG interface towards the vacuum interface, whose magnitude, $J_{\rm V} = |\mathbf{J}_{\rm V}|$, is proportional to $P_{\rm YIG} I_0$ due to the topological protection of the vortex number in the bulk. Analogously, under the current $+\mathbf{I}_0$, the vortex flow is generated in the opposite direction, $J_{\rm V} \propto P_{\rm vac} I_0$. Therefore, from the asymmetry of the nucleation rate [Supplementary Eq. (23)], we obtain the asymmetric vortex flow $\Delta J_{\rm V}$ under $-\mathbf{I}_0$ and $+\mathbf{I}_0$,

$$\Delta J_{\rm V} = J_{\rm V}(-I_0) - J_{\rm V}(+I_0) \propto (P_{\rm YIG} - P_{\rm vac})\, I_0 \propto \chi_\perp I_0. \tag{24}$$

Here, we consider the same mobility of vortex strings in both directions. Via the flux flow resistivity (Supplementary Ref. 8), the resultant vortex flow in the direction away from the YIG substrate towards the MoGe surface generates the negative polarity of voltage, which is consistent with the observed voltage signal in the MoGe|YIG sample

(setup shown in the inset of Fig. 3a of main text). The nonreciprocity is maximal in the vortex liquid phase, where the vortex mobility is the largest.

1.4 Consideration of sample length scales

So far, we have ignored the effect of geometry of the superconducting sample, for simplicity. Here, we consider the effect of the finite thickness of the MoGe film within our model for the vortex rectification.

The crucial aspect in the rectification mechanism discussed here is the nucleation of vortex semi-circles at the interfaces with a radius comparable to the coherence length $\xi \simeq \lambda_{\rm L}/100$, which at $T = 6$ K is roughly 20 nm, and that is much smaller than the thickness of the film used in this work, $t \simeq 350$ nm. The nucleated vortices are characterized by a core with the scale of coherence length ξ, and assumed to move freely without confinement or pinning, close to the resistive transition.

However, as for the magnetic flux, since the penetration depth $\lambda_{\rm L}$ is larger than the thickness, the magnetic field produced by the vortex is substantially extended beyond the MoGe film. As discussed in Sec. 1.2 of Supplementary Note 1, the difference of the vortex nucleation energy originates mainly in the transverse part of the magnetic coupling $F_{{\rm S}\perp}$, between the stray magnetic field produced by the nucleated vortex and the magnetization in the YIG substrate. Even though the actual magnetic field produced by the vortices in the vortex liquid in a thin film seems to be quite complicated, we assume that the stray magnetic field outside the film is approximately given by the dipole magnetic field [Supplementary Eq. (15)].

In order to discuss the effect of the finite thickness t of the MoGe film (Supplementary Fig. 1), we consider the dipole magnetic field which is emanating from the end points of the semi-circle vortex at $y = t$:

$$\mathbf{H}_t = -\boldsymbol{\nabla} U_t, \quad U_t = P\,\frac{z}{[x^2 + (y-t)^2 + z^2]^{3/2}}. \tag{25}$$

Using this dipole field, we can similarly evaluate the magnetic energy for a nucleated vortex at the MoGe/vacuum interface:

$$F_{{\rm S}\perp} = -\int_{y<0} d^3r\ \mathbf{H}_{t\perp} \cdot \delta\mathbf{M}_\perp \simeq -\chi_\perp\,\Phi_0^2\left(\frac{\rho_{\rm v}^2}{t^3}\right). \tag{26}$$

This guarantees that, for $t \gg \xi$, the nucleated vortex at the MoGe/vacuum interface has a negligibly small magnetic energy $\propto 1/t^3$, compared with the magnetic energy ($\propto 1/\xi^3$) of the vortex at the YIG/MoGe interface. Note that this property originates from the short-range potential for the dipole magnetic field, and not from the Meissner effect. Therefore, the difference of the vortex nucleation energy discussed so far is indeed

generated even in the case of the thin film, unless the thickness becomes smaller than the coherence length.

Similar argument can be applied to the case with the SiO_2 interlayer between the YIG and MoGe layers. In this case, neglecting the small (diamagnetic) susceptibility of SiO_2, the stray magnetic field emanating from the nucleated vortex at the SiO_2/MoGe interface feels the magnetization in the YIG substrate, separated by the SiO_2 interlayer with its thickness W. By a similar calculation, we can obtain the magnetic energy for the vortex, $F_{\mathrm{S}\perp} \propto -1/W^3$. This result implies that, for $W > \xi$, the magnetic energy rapidly decreases with the thickness W, and competes with the small diamagnetic contribution from the SiO_2 interlayer. The voltage due to vortex rectification is reduced with increasing thickness W, and is gradually replaced by the diamagnetic contribution.

Supplementary Note 2. Additional experimental results

2.1 Angular dependence of d.c. voltages

For measurements with different directions of the magnetic field, a PPMS rotator was used, where the samples were attached with varnish to the PPMS resistivity chip. The temperature of the chip was monitored with a Cernox thermometer. In order to accurately determine θ_H, the angle between the direction of the magnetic field and the normal to the sample plane, we have measured the angular dependence of the resistivity of MoGe at T and B fixed in the vortex liquid phase; a minimum in resistivity occurs just when the magnetic field is parallel to the sample plane (Supplementary Ref. 9). It is noted that for data shown in Figs. 2, 3 of the main text $\theta_H \approx 89°$, except for Fig. 3g,h,i where $\theta_H \approx 95°$.

Changing the angle of the magnetic field drastically modifies the amplitude of the d.c. voltage signal, as shown in Supplementary Fig. 3. The spontaneous voltage appears only when the field is almost parallel to the film plane ($| \theta_H - 90° | < 10°$). However, the voltage almost disappears when the field is just parallel to the film plane, $\theta_H \sim 90°$. Such anomalous behaviour was also confirmed in another MoGe|YIG sample with similar thickness (MoGe|YIG #2 in Supplementary Fig. 3c). For a magnetic field perpendicular to the film plane, the direction of the Lorentz force acting on the vortex strings is in the film plane. The vortex energy in this direction is symmetric and, therefore, the transport should be reciprocal. Only when the vortex strings are oblique to the film plane, the Lorentz force has a component in the direction perpendicular to the film plane. In this direction, the vortex energy is asymmetric between the interfaces (YIG/MoGe and MoGe/vaccuum) and facilitates the nonreciprocity observed as the d.c. voltage in Supplementary Fig. 3. On the other hand, as the field direction is becoming parallel to the film plane, voltage is rapidly suppressed. Although the mechanism is not clear yet, it is possible that this suppression may occur due to surface superconductivity (Supplementary Refs. 1, 9-17). If superconductivity appears only at the surfaces of MoGe, with the bulk remaining in normal state, vortex strings cannot traverse the bulk from one interface to the other. In such condition, the vortex flow would not be affected by the asymmetry in the nucleation energies at the two interfaces and the nonreciprocity would disappear. It is noted that the theoretical discussion of vortex flow in Supplementary Note 1 assumes that the whole volume of the sample is superconducting. In such case, large rectification effects would be expected even in magnetic fields parallel to the surface, and consideration of surface superconductivity could explain the observed suppression. An explanation of the observed vortex flow rectification solely due to surface superconductivity seems quite difficult, since surface

superconductivity destroys superconductivity in the bulk, and there is no vortex flow from interface to interface. The coherence length of electrons in the normal bulk of the film is too short and cannot feel the asymmetry between the surfaces.

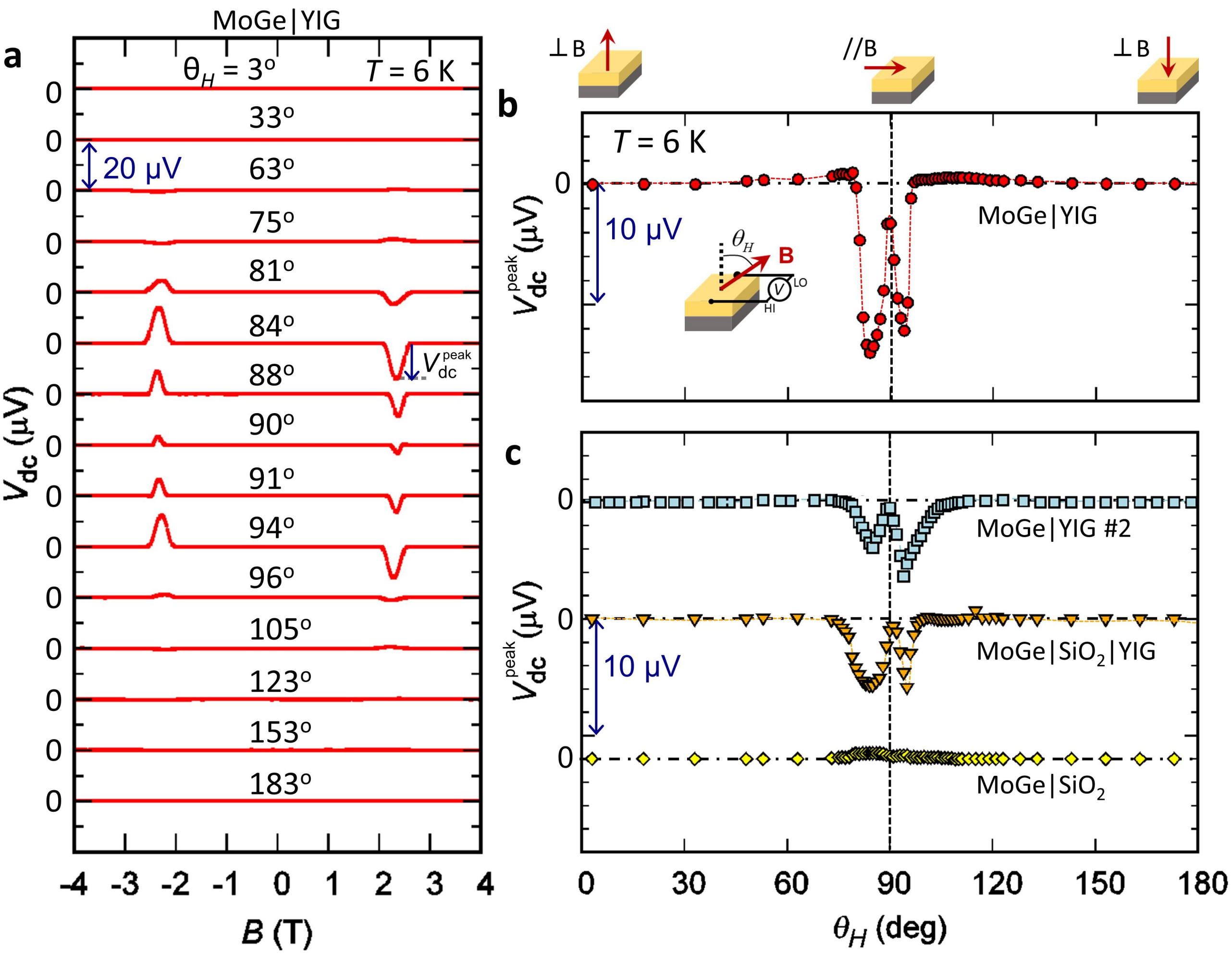

Supplementary Figure 3: Field-angular dependence of the d.c. voltage $V_{\rm dc}$ at $T = 6$ K. **a**, Magnetic field dependence of $V_{\rm dc}$ generated in MoGe|YIG at selected angles θ_H. Here, θ_H is the angle between the sample surface normal and the direction of the magnetic field. $V_{\rm dc}^{\rm peak}$ defines the peak value of $V_{\rm dc}$ at that angle. **b** and **c**, Field-angular dependence of $V_{\rm dc}^{\rm peak}$ in MoGe films on different substrates. Vertical dashed line shows the angle where the magnetic field direction is parallel to the film plane. Dotted curves are guides for the eye. Inset of **b** shows the definition of θ_H; yellow represents the MoGe layer and grey represent the substrate.

2.2 Estimate of the d.c. voltage generated by rectification of environmental noise

Supplementary Figure 4 shows an energy density spectrum of the environmental electromagnetic fluctuations measured with a coil inserted into the sample space of the PPMS system. The estimated dominant contribution is in the 2 - 4 MHz range. The integrated noise energy density in the sample space, to which the sample is exposed, is 40 μJm^{-3}. This corresponds to typical magnetic field fluctuations $\delta B = 10$ μT. The noise current induced in the sample through its inductance $L = 3$ μH is $I_0 = \delta B S_{\mathrm{surf}}/L = 40$ μA, where S_{surf} is the surface area of the sample. Then, using $\Delta_{2f} = -0.4 \times 10^{-3}$ ΩA^{-1}.m (see Fig. 2g at $T = 6$ K in main text), the expected d.c. voltage generated in the MoGe|YIG is $V_{\mathrm{dc}} = \frac{1}{2}\Delta_{2f} I_0^2 \frac{\ell}{S} = -10^{-6}$ V, where S is the cross-section area of the MoGe film, and ℓ is the distance of the electrodes. This rough estimate gives a V_{dc} of the same order as the value obtained in the d.c. voltage measurements.

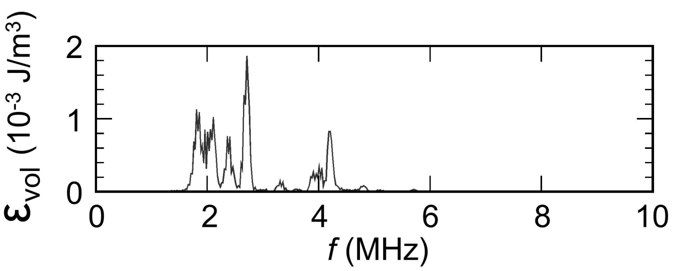

Supplementary Figure 4: Energy density spectrum ϵ_{vol} of electromagnetic environmental noise in the sample space.

We also discuss the conversion efficiency in our system, defined as $P_{\mathrm{out}}/P_{\mathrm{in}}$, where P_{out} is the generated power, and P_{in} is the irradiated power. The irradiated spectrum has a maximum at 3 MHz with $P_{\mathrm{in}} = -40$ dBm (10^{-7} W, Supplementary Fig. 4), and with a load of 10 Ω, we have $P_{\mathrm{out}} = 10^{-13}$ W at $T = 6$ K, and $B = 2.5$ T (Fig. 3c in main text). This corresponds to an upper bound for power conversion efficiency of 10^{-6}.

The known rectenna devices have conversion efficiencies dependent on operating frequency, as shown in Supplementary Table 1. The efficiencies range from 15% to 92% in the microwave region, with the lowest reported frequency being 430 MHz. In the infrared, visible and UV region, the only reported rectenna we are aware of has a conversion efficiency of 10^{-5} (Supplementary Ref. 22). While our conversion efficiency is small compared to those in the microwave region, it is comparable to that in the infrared region.

Supplementary Table 1: Experimentally reported conversion efficiencies of rectenna devices.

	Operating frequency	Conversion Efficiency	Supplementary Reference
Radio Waves	3 MHz	10^{-6}	this work
Microwave	430 MHz	86%	[18]
	2.3 GHz $\sim$ 100 GHz	15 $\sim$ 92%	[19, 20, 21]
Infrared, Visible, UV	30 THz	10^{-5}	[22]

2.3 Temporal evolution of d.c. voltage in MoGe|YIG with superconducting magnet in persistent mode.

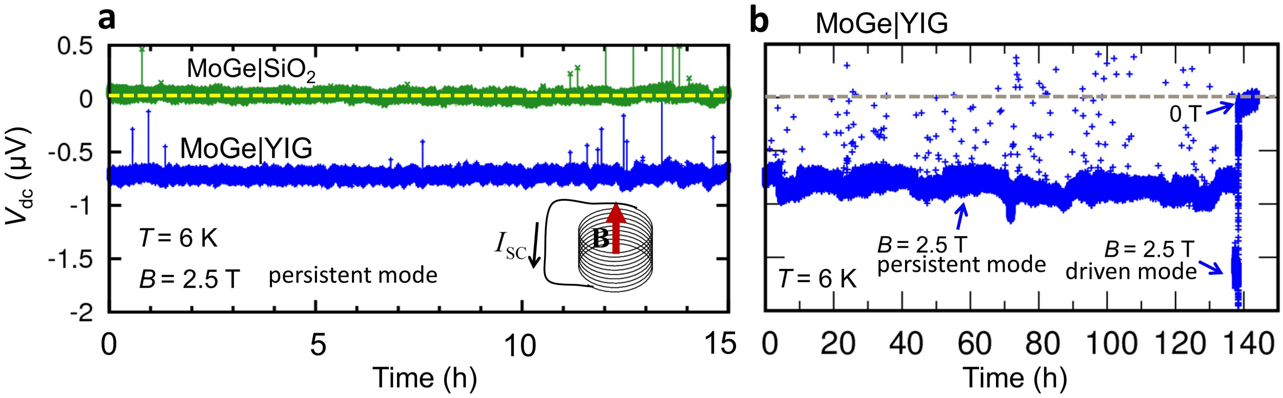

Supplementary Figure 5: **a**, Temporal evolution of the d.c. voltage V_{dc} in MoGe|YIG (blue) and MoGe|SiO_2 (green) during 15 hours at $T = 6$ K and magnetic field $B = 2.5$ T with the superconducting magnet set in persistent mode. The d.c. voltages are constant during the entire observation. This shows that the observed d.c. voltage is not a transient phenomenon but is generated steadily as long as MoGe is kept in the vortex liquid phase. **b**, Temporal evolution of V_{dc} in MoGe|YIG during 140 hours at $T = 6$ K and magnetic field $B = 2.5$ T. The d.c. voltage is constant as long as the magnet is kept in persistent mode. At the end of a 135-hour observation, the magnet is switched to driven mode which is accompanied by a rise in the d.c. voltage. When the magnetic field is set to 0 T (i.e. outside of the vortex liquid phase) at the 140 hours mark, the d.c. voltage vanishes.

2.4 Change in d.c. voltage generated in MoGe|YIG by switching of the superconducting magnet between driven and persistent modes.

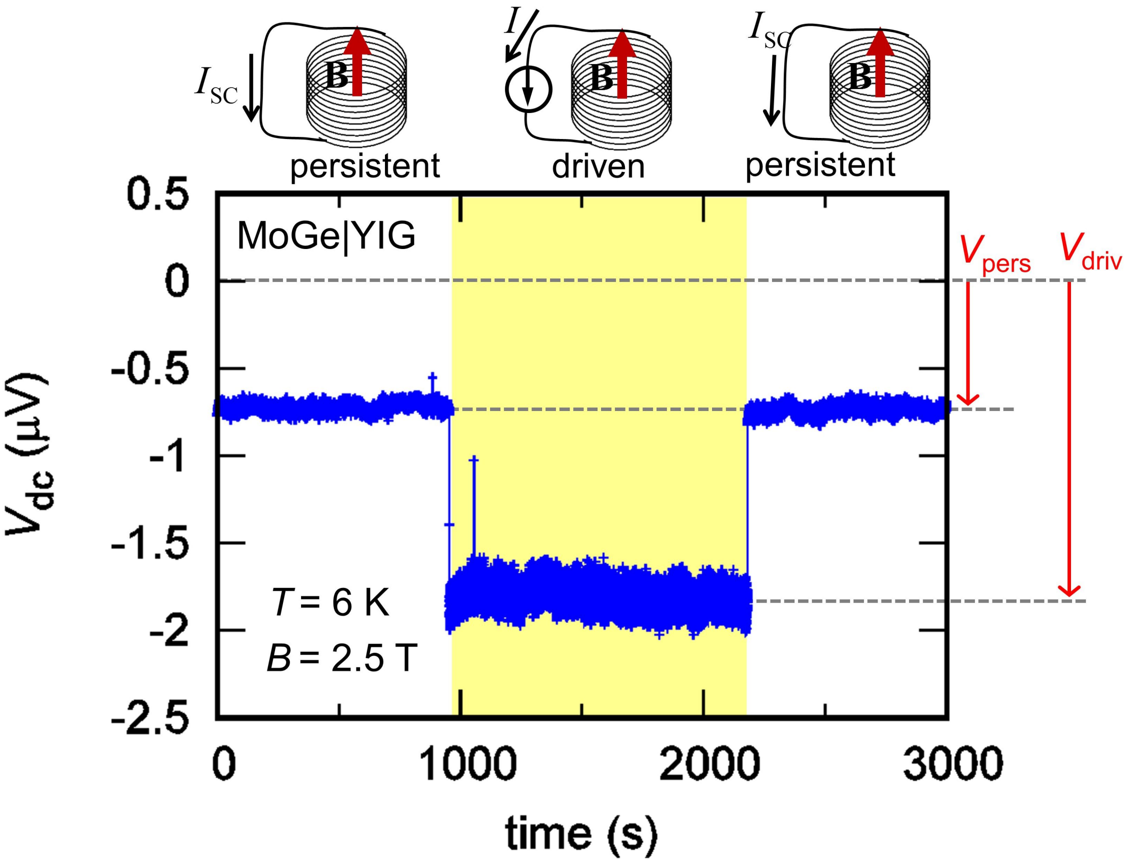

Supplementary Figure 6: Change in the d.c. voltage V_{dc} in MoGe|YIG caused by switching of the superconducting magnet between persistent and driven mode at $T = 6$ K and $B = 2.5$ T. The magnet is first kept in persistent mode for 1000 seconds. The generated d.c. voltage is constant, $V_{pers} = -0.7$ μV. Then, the magnet is switched to driven mode and kept for 1200 seconds (yellow region). In driven mode, the power supply is directly connected to the superconducting coil and the fluctuations in the magnet power supply lead to an increase in environment fluctuations inside the sample space. In response, the d.c. voltage increases to $V_{driv} = -1.8$ μV, and stays constant while the magnet is kept in driven mode. At time = 2200 seconds, when the magnet is switched to persistent mode again, the d.c. voltage drops back to the V_{pers} value immediately, and stays constant thereafter.

2.5 Magnetic field dependence of d.c. voltages generated in MoGe|YIG for various in-plane temperature gradients.

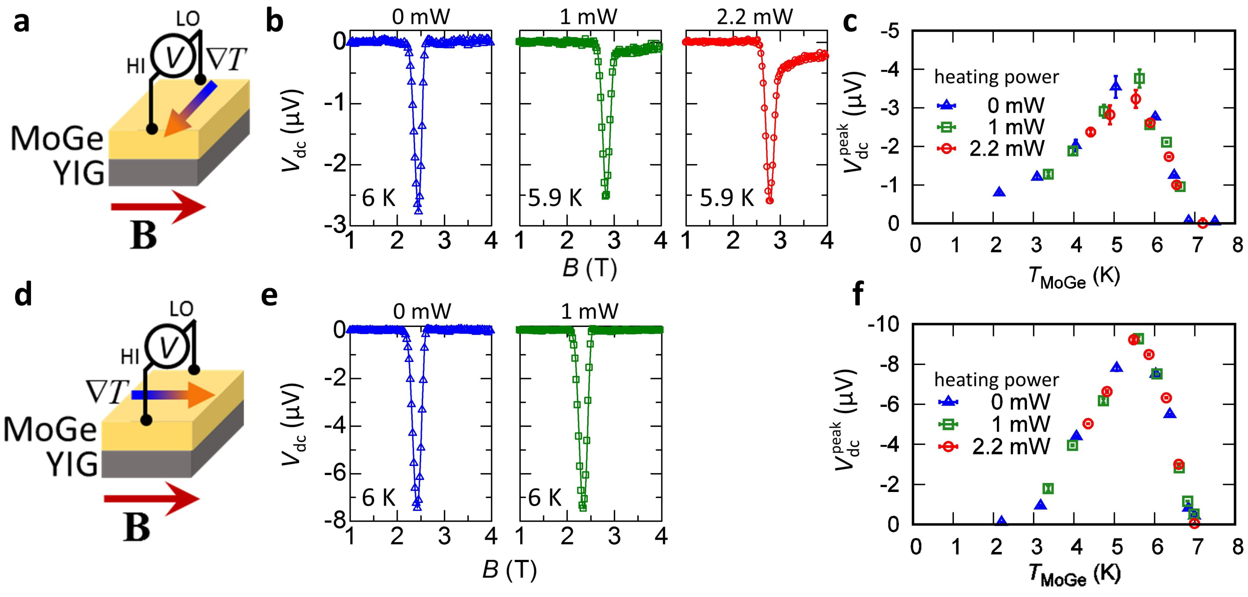

Supplementary Figure 7: **a** (**d**), A schematic illustration of the measurement setup with the temperature gradient ∇T in the film plane perpendicular (parallel) to the magnetic field **B** direction. **b** (**e**), Magnetic field dependence of d.c. voltages V_{dc} observed in the MoGe|YIG sample for selected heating powers, 0 mW, 1 mW and 2.2 mW, with in-plane temperature gradients perpendicular (parallel) to the magnetic field. The temperature of the MoGe film is constant (5.9 $\sim$ 6 K). The peak value of the d.c. voltage $V_{\mathrm{dc}}^{\mathrm{peak}}$ does not change with ΔT, showing that the d.c. voltage generation is not affected by in-plane temperature gradients. **c** (**f**), Temperature dependence of the peak value of the d.c. voltage $V_{\mathrm{dc}}^{\mathrm{peak}}$ for selected values of heating power with in-plane temperature gradient perpendicular (parallel) to the magnetic field direction. The heating power applied is irrelevant to the observed temperature dependence, which shows that in-plane temperature gradients are not related to the spontaneous d.c. voltage generation. Error bars correspond to the background noise.

2.6 Magnetic field dependence of the resistivity, Δ_{2f}, and generated d.c. voltages in MoGe|SiO_2(10 nm)|YIG at selected temperatures.

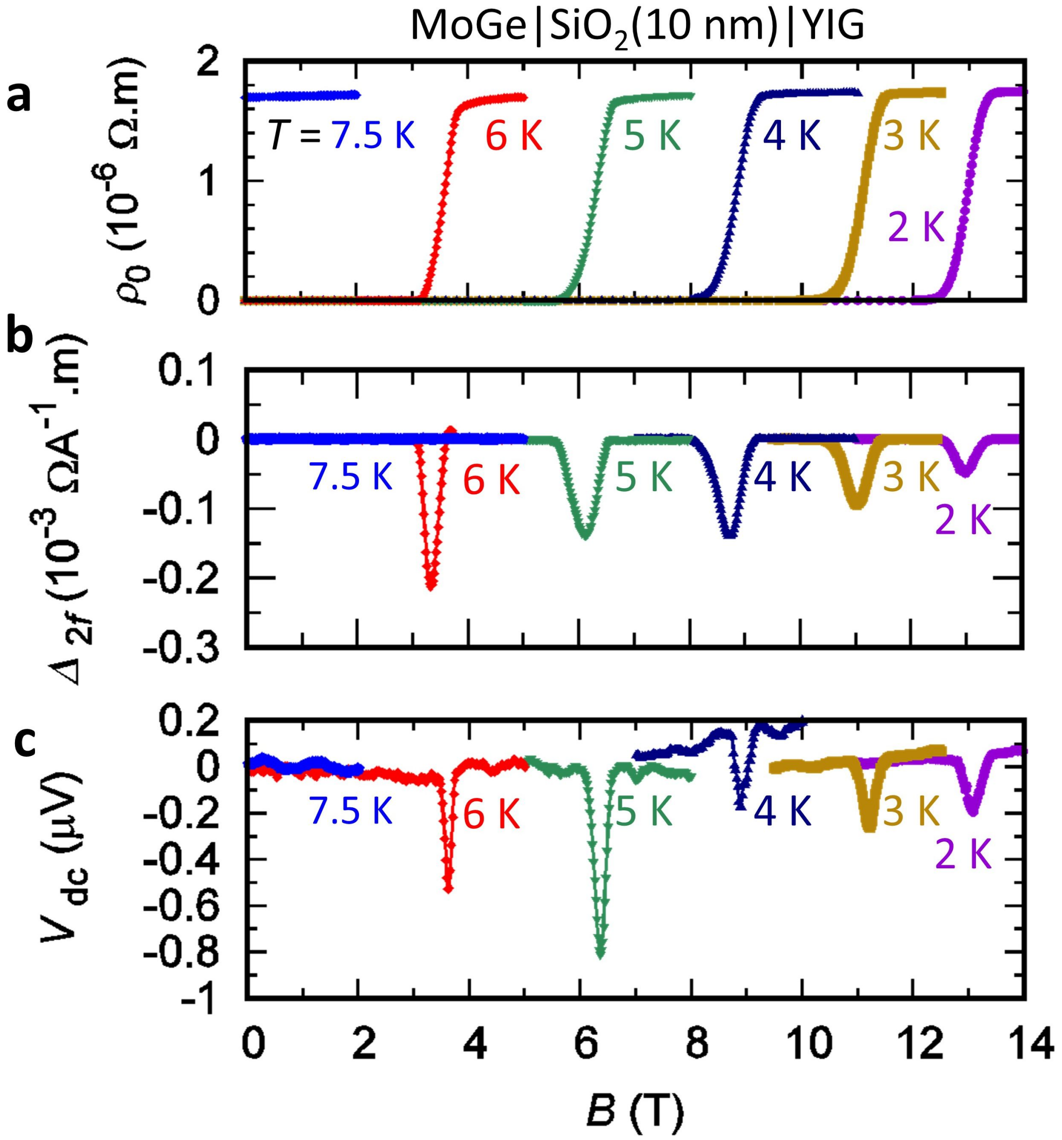

Supplementary Figure 8: Magnetic field dependence of **a**, the resistivity ρ_0, **b**, the nonreciprocal resistivity coefficient Δ_{2f}, and **c**, generated d.c. voltages V_{dc} in MoGe|SiO_2(10 nm)|YIG at selected temperatures. Peaks in Δ_{2f} and peaks in V_{dc} appear at each temperature at the resistive transition of MoGe, consistent with the observation in MoGe|YIG. The size and sign of the observed Δ_{2f} and V_{dc} are comparable to those observed in MoGe|YIG (see Figs. 2g and 3b in the main text).

2.7 Comparison of d.c. voltages generated in MoGe|SiO_2, MoGe|$Y_3Al_5O_{12}$(YAG) and MoGe|YIG

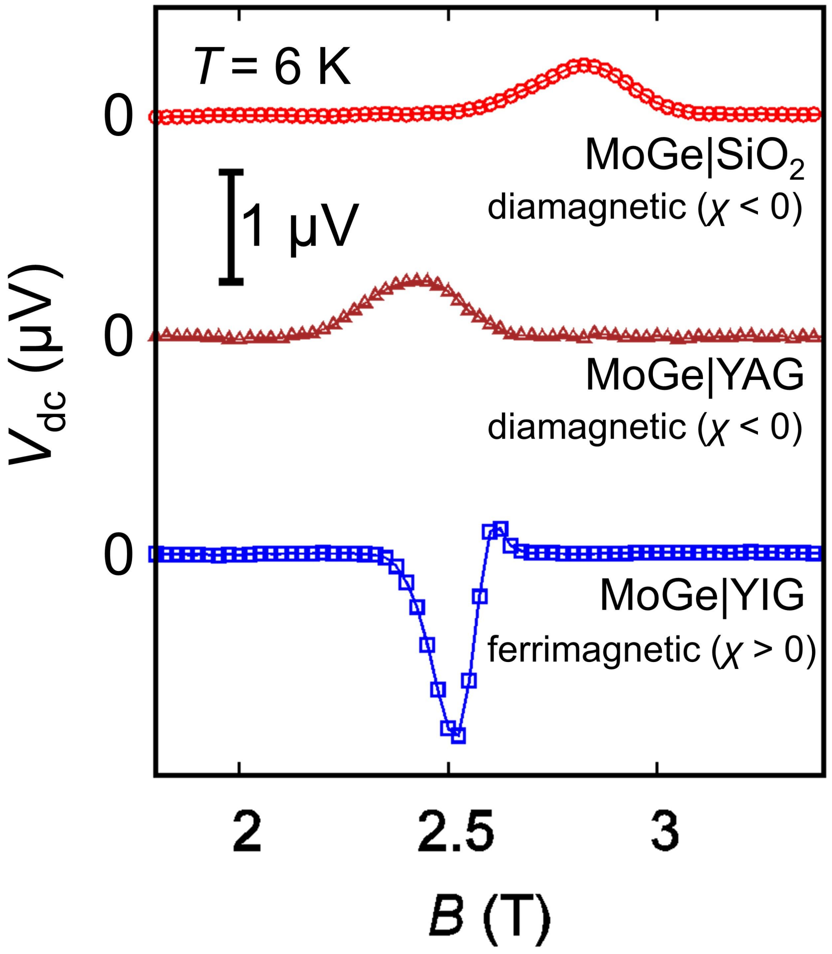

Supplementary Figure 9: Magnetic field dependence of d.c. voltages V_{dc} generated in MoGe|SiO_2 (red circles), MoGe|$Y_3Al_5O_{12}$ (YAG, brown triangles) and MoGe|YIG (blue squares) at $T = 6$ K. YAG has a garnet structure similar to YIG and is diamagnetic like SiO_2. The d.c. voltages are of positive polarity on the SiO_2 and YAG substrates, which are diamagnetic ($\chi < 0$), and negative on the YIG substrate, which is ferrimagnetic ($\chi > 0$). This shows that the polarity of the generated d.c. voltage is determined by the magnetic properties of the substrate, not its morphology.

2.8 Magnetic field dependence of d.c. voltages in MoGe|YIG at temperatures above 6 K

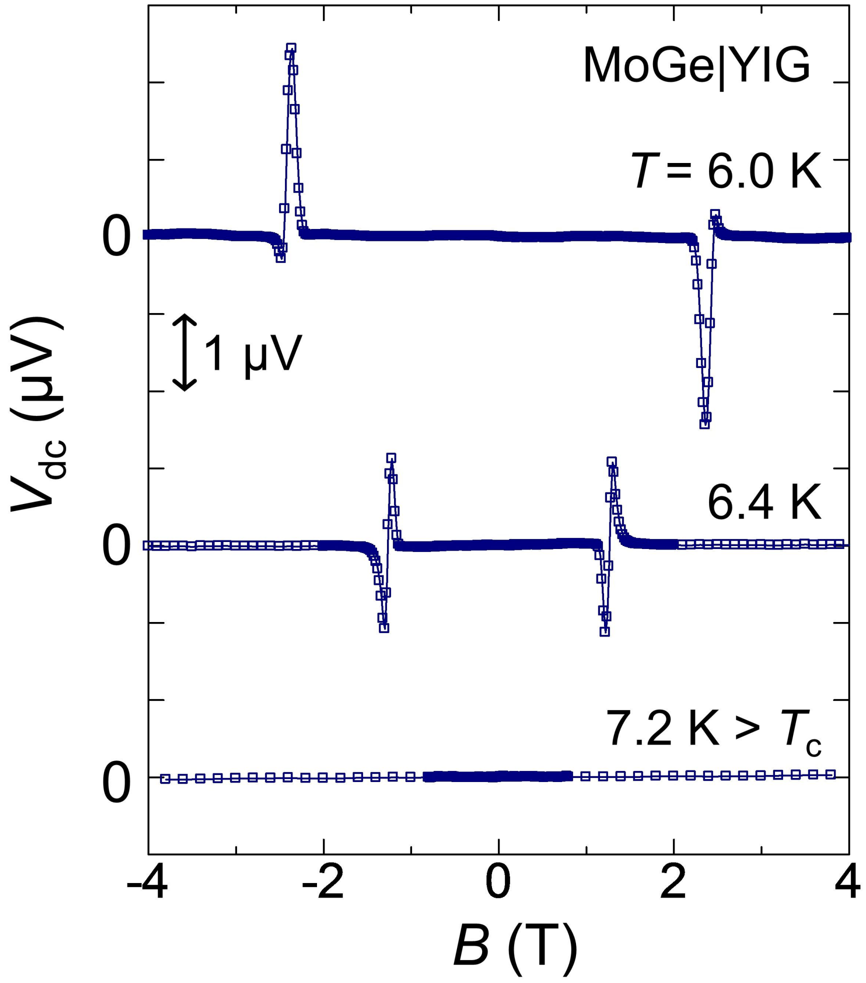

Supplementary Figure 10: Magnetic field dependence of d.c. voltages V_{dc} generated in MoGe|YIG at T = 6.0 K, 6.4 K, and 7.2 K ($> T_{\mathrm{c}}$). At higher temperatures, where the superconducting transition is approaching zero field, the amplitude of the signal becomes smaller. This decrease might be caused by a decrease in the vortex density with decreasing magnetic field, and/or due to increasing probability of antivortex nucleation, which would cancel out the vortex contribution. We note that the positive spike in d.c. voltage spectrum, which appears prominently at higher temperatures, is attributed to Nernst effects due to an accidental temperature gradient; the voltage spectrum has the same shape as that for $\Delta T > 0$ in Fig. 3d of main text.

2.9 Influence of SiO_2 thickness on d.c. voltages in MoGe|SiO_2|YIG

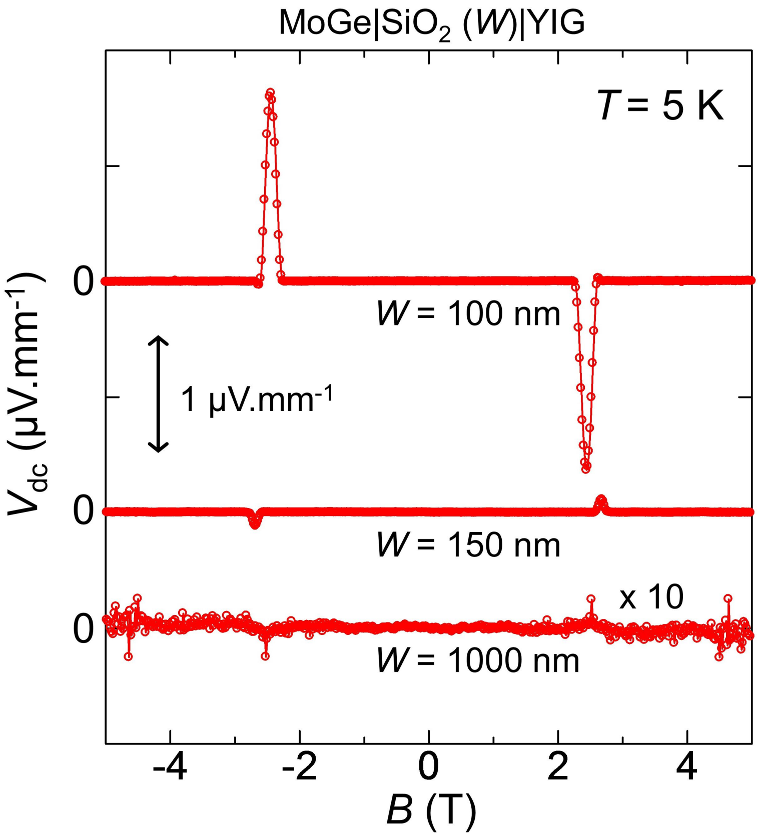

Supplementary Figure 11: D.c. voltages V_{dc} generated in MoGe|$SiO_2(W)$|YIG for varying thickness of SiO_2 ($W = 100$, 150, and 1000 nm) at $T = 5$ K. For $W = 100$ nm, the d.c. voltage is still present. For $W = 150$ nm, the negative d.c. voltage is not observed and instead a small positive voltage appears, similar to films on oxidized Si substrates. This trend is reproduced in a sample with $W = 1000$ nm (plotted 10 times larger for increased visibility). This suggests that the magnetic coupling between YIG and vortices nucleating in MoGe has a length scale of the same order as the coherence length ξ. We note that the SiO_2 films were sputtered at room temperature and are amorphous. As such, their density is not controlled and they are likely of different quality than the oxidized Si substrates. Therefore, a discussion about the amplitude of the d.c. voltages is not reasonable, since this is also affected by the amplitude of the magnetic susceptibility in the substrate. All d.c. voltages were normalized by the distance between electrodes because the samples used in this measurement were of different size. The T_c in these films is 6 K.

2.10 Effect of the shape and size of electric contacts on d.c. voltages in MoGe|YIG

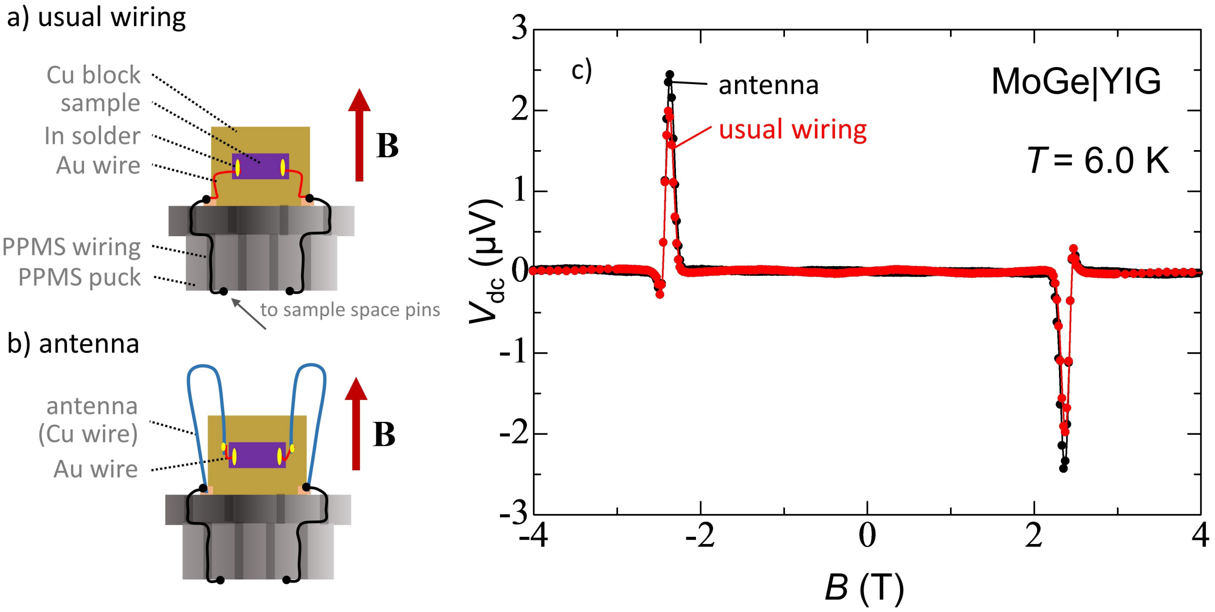

Supplementary Figure 12: Role of sample contacts in the ambient radiaton pickup of our rectenna device. a), b) Schematic illustration of the sample setup. In a) Au wires are attached to the sample with In solder, which is the usual wiring used in all remaining measurements. In b) a bent copper wire (antenna) with a diameter of 0.20 mm and a length of 30 mm is added in order to verify the antenna property of the sample contacts. c) The magnetic field dependence of d.c. voltages V_{dc} in MoGe|YIG in both cases. When the contacts are altered to enhance their antenna property, the increase in the d.c. voltage amplitude is less than 18%, which is negligible compared to the variation observed among samples on different substrates (almost 90%, see Fig. 3g-i in main text). We speculate that the most significant part contributing to the radiation pickup is the chamber wiring of the PPMS cryostat, which is coupled by a capacitance of roughly 100 pF to the cryostat ground (Supplementary Ref. 23). The a.c. currents running through the chamber wiring, and consequently through the sample, are then rectified to the observed d.c. voltage (see also Supplementary Figs. 13-15).

2.11 Effect of shielding and grounding on d.c. voltages in MoGe|YIG

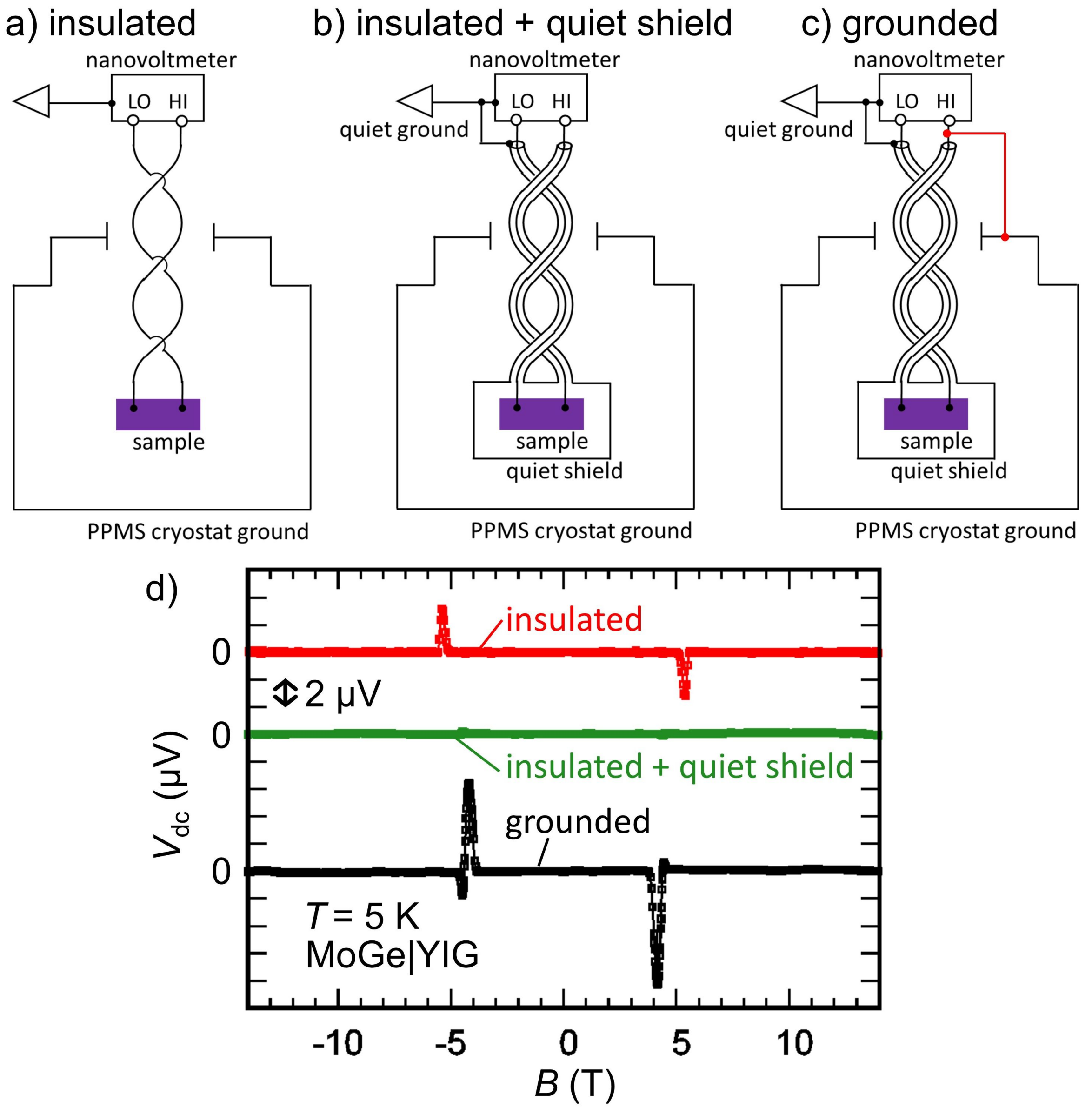

Supplementary Figure 13: Influence of coupling of the sample wiring to cryostat ground on the d.c. voltage V_{dc} generation in MoGe|YIG. a) A schematic illustration of the d.c. voltage measurement configuration for the data in the main text (Fig. 3) and in the remaining Supplementary Figures in Supplementary Note 2. The sample and wiring are insulated from the cryostat ground ("insulated"). b), c) Schematic illustrations of control experiments. To prove the speculation that a.c. currents in the sample appear due to coupling of wiring to the PPMS ground, a custom setup was constructed using a functional rod in which the sample is wired with an external coaxial twist-pair cable and surrounded by a shield. In b), the shield and the sample wiring are insulated from the PPMS ground and the shield is connected to a quiet ground of the nanovoltmeter

(“insulated + quiet shield”). In c), the sample wiring is directly connected to the PPMS cryostat ground shown as a red wire (“grounded”). d) Magnetic field dependence of the d.c. voltages at $T = 5$ K. The d.c. voltage from Fig. 3 in the main text is shown with red points (“insulated” configuration). When the sample and wiring are completely shielded with a quiet ground (“insulated + quiet shield” configuration, green points), the d.c. signal almost vanishes. On the other hand, when one electrode of the sample is connected to the PPMS cryostat ground (“grounded” configuration, black points), a large d.c. voltage (almost 10 μV) is observed. Thus, the wiring picks up mainly electric noise from the cryostat ground, which can be responsible also for d.c. voltage generation in the insulated configuration a). It is noted that the slight shift in the peak position in c) compared to a) might be due to a temperature shift caused by the use of a custom sample probe.

2.12 Influence of the magnet power supply noise on the d.c. voltage in MoGe|YIG

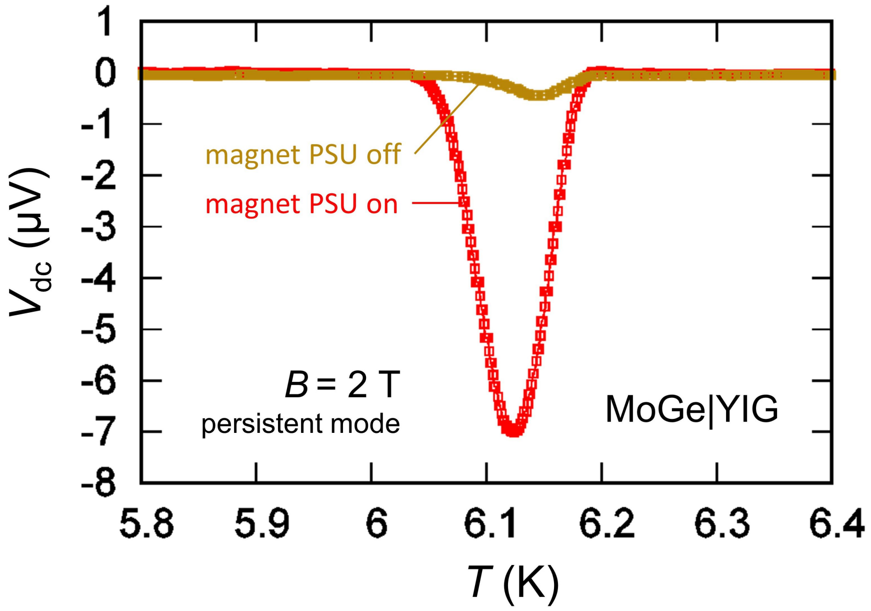

Supplementary Figure 14: Influence of the power mode of the magnet power supply unit (PSU) on the d.c. voltages V_{dc} generated in MoGe|YIG. The magnetic field is set to $B = 2$ T in persistent mode. Temperature control is turned off and the temperature of the sample is naturally increasing. Red points show V_{dc} in the usual measurement condition with the magnet PSU powered on. Brown points show V_{dc} for the magnet PSU powered off. The d.c. voltage almost vanishes when PSU is powered off. The on/off difference is likely caused by a standby current in PSU leaking into the cryostat ground and increasing the noise level when powered on. This indicates that the MoGe|YIG measurement system can pick up the low-level current noise in cryostat ground generated by the magnet PSU (see also Supplementary Figs. 13 and 15).

2.13 Diagram of the rectenna device

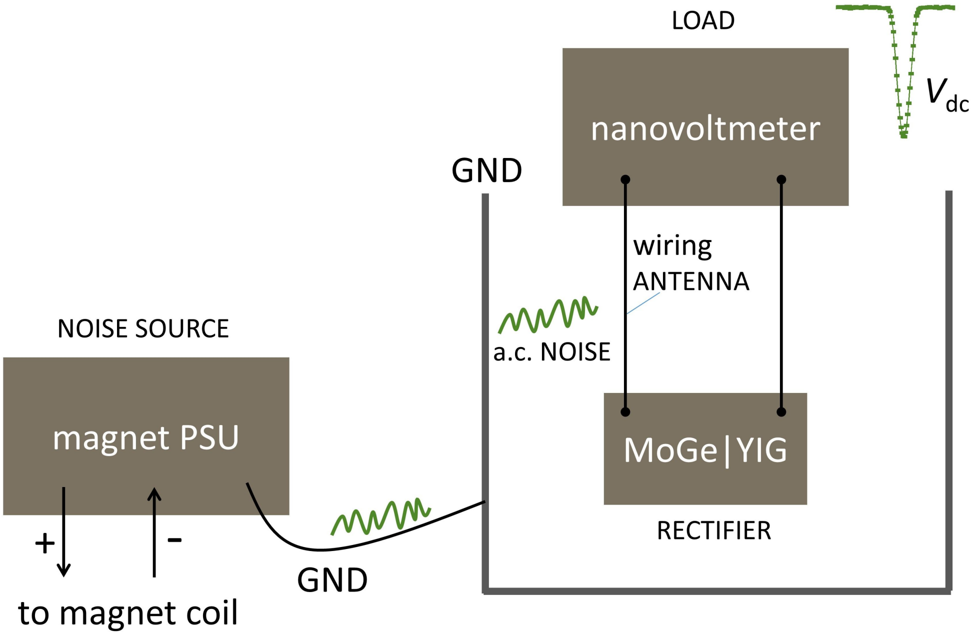

Supplementary Figure 15: Schematic illustration of our rectenna device. The magnet power supply unit (PSU) is a noise source (Supplementary Fig. 14), from which electric field fluctuations leak into the cryostat ground (GND). The wiring of the cryostat chamber acts as an antenna that picks up fluctuations of an electric field from the cryostat through the insulation (Supplementary Fig. 13). The MoGe|YIG sample works as a rectifier and generates d.c. voltages from the a.c. noise current, which are detected at the nanovoltmeter.

Supplementary References

1. Tinkham, M. *Introduction to superconductivity.* (Courier Corporation, 1996)
2. Samokhvalov, A.V. Vortex loops entry into type-II superconductors. *Phys. C: Superconductivity*, **259**, 337-348 (1996).
3. Petukhov, B.V. & Chechetkin, V.R. Rate of penetration of a magnetic flux into type-II superconductors. *Zh. Eksp. Teor. Fiz.* **65**, 1653-1657 (1973).
4. Ochoa, H., Kim, S.K., & Tserkovnyak, Y. Topological spin-transfer drag driven by skyrmion diffusion. *Phys. Rev. B* **94**, 024431-1-7 (2016).
5. Bean, C.P. & Livingston, J.D. Surface barrier in type-II superconductors. *Phys. Rev. Lett.* **12**, 14-16 (1964).
6. Halperin, B.I., Refael, G., & Demler, E. Resistance in superconductors. *BCS: 50 Years*, pp. 185-226 (World Scientific, 2010).
7. Takahashi, S. & Maekawa, S. Spin current in metals and superconductors. *J. Phys. Soc. Jpn.* **77**, 031009-1-14 (2008).
8. Huebener, R.P. *Magnetic flux structures in superconductors*, 2nd ed. (Springer, 2001).
9. Monceau, P., Saint-James, D., & Waysand, G. Studies of parallel and tilted vortices in type-II superconductors by microwave absorption. *Phys. Rev. B* **12**, 3673-3689 (1975).
10. Saint-James, D. & deGennes, P.G. Onset of superconductivity in decreasing fields. *Phys. Lett.* **7**, 306-308 (1963).
11. Hempstead, C.F. & Kim, B.J. Resistive transitions and surface effects in type-II superconductors. *Phys. Rev. Lett.* **6**, 145-148 (1964).
12. Yamafuji, K., Kusayanagi, E. & Irie, F. On the angular dependence of the surface superconducting critical field. *Phys. Lett.* **21**, 11-13 (1966).
13. Yamafuji, K., Kawashima, T. & Irie, F. On the angular dependence of the upper critical field in thin films. *Phys. Lett.* **20**, 122-123 (1966).
14. Monceau, P. & Gilchrist, J.G. Resistive behavior of superconducting surface sheats of alloys. *J. Low Temp. Phys.* **5**, 363-375 (1971).
15. Chin, C.C. & Orlando, T.P. Angular dependence of H_{c3} for an anisotropic superconductor with proximity effect. *Phys. Rev. B* **37**, 5811-5813 (1988).

16. Pan, A.V. & Esquinazi, P. Decoupling transition of two coherent vortex arrays within the surface superconductivity state. *Phys. Rev. B* **70**, 184510-1-6 (2004).

17. Xie, H.Y., Kogan, G.V., Khodas, M., & Levchenko, A. Onset of surface superconductivity beyond the Saint-James-de Gennes limit. *Phys. Rev. B* **96**, 104516-2-15 (2017).

18. Huang, F.-J., Lee, C.-M., Chang, C.-L., Chen, L.-K., Yo, T.-C., & Luo, C.-H. Rectenna application of miniaturized implantable antenna design for triple-band biotelemetry communication. *IEEE Trans. Antennas Propag.* **59**, 2646-2653 (2011).

19. Brown, W.C. The history of power transmission by radio waves. *IEEE Trans. Microwave Theory Tech.* **32**, 1230-1242 (1984).

20. Brown, W.C. Optimization of the efficiency and other properties of the rectenna element. *Microwave Symp., IEEE MTT-S Int.*, 1976, pp.142-144.

21. Donchev, E. *et. al*, The rectenna device: From theory to practice (a review), *MRS Energy and Sustainability.* doi:10.1557/mre.2014.6

22. Sharma, A., Singh, V., Bougher, T.L., & Cola, B.A. A carbon nanotube optical rectenna. *Nature Nanotechnology* **10**, 1027-1032 (2015).

23. PPMS and DynaCool Sample Chamber Info for Users, Quantum Desing, Inc.
